# Effects of Vocal Fold Nodules on Glottal Cycle Measurements Derived from High-Speed Videoendoscopy in Children

**DOI:** 10.1371/journal.pone.0154586

**Published:** 2016-04-28

**Authors:** Rita R. Patel, Harikrishnan Unnikrishnan, Kevin D. Donohue

**Affiliations:** 1 Department of Speech & Hearing Sciences, Indiana University, Bloomington, Indiana, United States of America; 2 Department of Electrical and Computer Engineering, University of Kentucky, Lexington, Kentucky, United States of America; Sun Yat-sen University, CHINA

## Abstract

The goal of this study is to quantify the effects of vocal fold nodules on vibratory motion in children using high-speed videoendoscopy. Differences in vibratory motion were evaluated in 20 children with vocal fold nodules (5–11 years) and 20 age and gender matched typically developing children (5–11 years) during sustained phonation at typical pitch and loudness. Normalized kinematic features of vocal fold displacements from the mid-membranous vocal fold point were extracted from the steady-state high-speed video. A total of 12 kinematic features representing spatial and temporal characteristics of vibratory motion were calculated. Average values and standard deviations (cycle-to-cycle variability) of the following kinematic features were computed: normalized peak displacement, normalized average opening velocity, normalized average closing velocity, normalized peak closing velocity, speed quotient, and open quotient. Group differences between children with and without vocal fold nodules were statistically investigated. While a moderate effect size was observed for the spatial feature of speed quotient, and the temporal feature of normalized average closing velocity in children with nodules compared to vocally normal children, none of the features were statistically significant between the groups after Bonferroni correction. The kinematic analysis of the mid-membranous vocal fold displacement revealed that children with nodules primarily differ from typically developing children in closing phase kinematics of the glottal cycle, whereas the opening phase kinematics are similar. Higher speed quotients and similar opening phase velocities suggest greater relative forces are acting on vocal fold in the closing phase. These findings suggest that future large-scale studies should focus on spatial and temporal features related to the closing phase of the glottal cycle for differentiating the kinematics of children with and without vocal fold nodules.

## Introduction

Mechanical trauma on vocal fold tissues resulting from vocal hyperfunction has an important role in the pathogenesis of vocal nodules. [1, 2]. Vocal nodules occur in 38–78% [3–5] of children and are thought to result from mechanical influences of vocal hyperfunction [1, 2] resulting in chronic hoarseness in 2% [6] to 23.4% [4] of children. In the treatment seeking population vocal nodules accounted for 63% of children in the age range of 0–14 years [7]. Dysphonia can be detrimental to children both psychologically [8, 9] and academically [10]. Hence early identification of dysphonia in children is critical. Evaluation of dysphonia is multidimensional involving a battery of tests, like acoustics, aerodynamics, perceptual evaluation, outcome measurements, and laryngeal imaging. Accurate evaluation of the cause of dysphonia is typically dependent on assessment of vocal fold structure and the resulting vibratory motion through techniques of laryngeal imaging.

Vibratory function and biomechanics of vocal hyperfunction and high impact stress leading to the development of nodules are largely determined by length of the membranous portion of the vocal folds and the stiffness. Biomechanical modeling of adults and animal models using excised larynges have hypothesized that high impact stress is related to short vocal fold length [11], increased amplitude [11, 12], high vocal fold closing velocity [13], increased peak vocal fold acceleration [1, 2], stiffness [12], and increased contact duration at the site of the nodules [1, 14]. These factors reportedly have an important role in the pathophysiology of formation of vocal nodules. Unfortunately, there is dearth of empirical knowledge regarding these laboratory findings for clinical practice, especially for normal and disordered pediatric voice.

Limited investigations from acoustic and aerodynamic analysis reveal indirect evidence of vocal hyperfunction behavior in children with vocal fold nodules. Hufnagle (1982) [15] reported increased fundamental frequency in 13 children with vocal fold nodules when compared to children without nodules from acoustic analysis of sustained phonation on the vowel /a/; suggesting increased tension. Leeper (1976) [16] reported increased airflow volume (amount of expired air during the first 200 millisecond of phonation) during ‘hard’ voice onset in children with vocal fold nodules compared to children with normal voice, suggesting deleterious effects of added vocal fold mass for children with vocal fold nodules. These findings though valuable only provide indirect inferences regarding vocal fold vibratory function. To the best of our knowledge, investigations into the effects of nodules on vocal fold vibrations through techniques of laryngeal imaging are lacking in children. In this study, we propose to quantify the effects of vocal fold nodules on vocal fold vibrations in pre-pubertal children with the use of high-speed videoendoscopy and normalized kinematic features that quantify the opening and closing phases of the glottal cycle [17].

In the pediatric population high-speed videoendoscopy with increased temporal resolution appears to be ideal for qualitative [18] and quantitative [19, 20] assessment of vocal fold vibratory function. With increased temporal resolution, recordings from high-speed videoendoscopy provide an opportunity to quantify individual vocal fold movements and derive kinematic correlates of mechanical influences of vocal hyperfunction and high impact stress; known to have an important role in the pathogenesis of vocal nodules [1, 2, 21, 22]. Stroboscopy, though the current gold standard in laryngeal imaging of vocal fold vibrations in adults, is limited for studying normal and disordered phonation in the pediatric population because of the limited temporal resolution [18, 23]. Stroboscopy requires a fairly regular vibration of the vocal folds for at least 3–4 seconds before the strobe light can track phonation [24]. In children it is often difficult to obtain phonation samples of greater than 2–3 seconds [25] required for stroboscopic recording due to variability in attention span and participation, resulting in non-interpretable findings from instrumental artifacts.

Several investigators have reported differences in vibratory motion in adults with vocal fold nodules using high-speed videoendoscopic imaging. Investigations in one female with vocal fold nodules revealed anterior-posterior phase delay with high values of maximum vocal fold velocity and accelerations particularly in the closing phase of the glottal cycle representing high vocal fold collisions [26]. Using digital kymographic analysis Chodara et al (2012) revealed that the group with vocal fold nodules (n = 17 females) demonstrated reduced lateral phase difference and amplitude symmetry compared to the control group (males = 19, females = 17) and the group with vocal fold polyps (males = 20, females = 14), with nodules showing more symmetrical vibratory motion compared to vocal fold polyps [27]. Similarly, Yamauchi et al (2015) revealed greater lateral phase difference, smaller mucosal wave, and reduced glottal closure in 6 individuals with vocal fold nodules [28]. In yet another study Krausert et al (2012) demonstrated statistical significance in spatiotemporal features of correlation length and entropy between the control (n = 67) and the pathological group (nodules plus polyps = 57), however these features were not statistically significant between nodules (n = 20) and polyps (n = 37) in this study [29]. To the best of our knowledge we were not able to locate similar studies investigating vibratory differences in children with vocal fold nodules. It is critical to evaluate the nature of disturbance in the vibratory motion due to the presence of vocal fold nodules in children, since there are known differences not only in laryngeal anatomy [30–32] but also in vocal fold vibratory function [17, 18, 20, 33–35].

Findings from normalized kinematic features of vocal fold displacements revealed that vibratory motion in typically developing children is generally larger compared to adult males and females. Children exhibited larger normalized peak displacement compared to adult males and females [17]. Greatest differences were obtained for the closing phase of the glottal cycle compared to the opening phase. Typically developing children demonstrated large values for normalized average closing velocity, normalized peak closing velocity, and normalized peak closing acceleration in addition to the speed quotient compared to adult males and females [17]. The current study uses these features of vocal fold displacement extracted from the mid-membranous point of the vocal fold [17] to assess the impact of vocal fold nodules on the opening and closing phases of the glottal cycle in children. The purpose of this study was to assess the effects of vocal fold nodules on vibratory kinematic parameters in children to establish the clinical utility of high-speed videoendoscopy and the basis for future research in this understudied population.

## Methods

### Participants

A total of 40 children in the age range of 5–11 years were recruited for this study after signing of IRB approved informed consent and assent forms. The Social/Behavioral/Educational full review board, Indiana University and the Office of Research Integrity Expedited Review Board at the University of Kentucky approved the study. Written informed consent from the next of kin, caretakers, and/or guardians on behalf of the minors/children enrolled in the study was obtained on IRB approved consent and assent forms. Of the total 20 children with vocal fold nodules, 5 were recruited at the University of Kentucky and 15 were recruited at the Vocal Physiology and Imaging Laboratory, Indiana University. Age and gender matched control subjects (n = 20) without any voice disorder were recruited at the Vocal Physiology and Imaging Laboratory, Indiana University.

**Fig 1:**
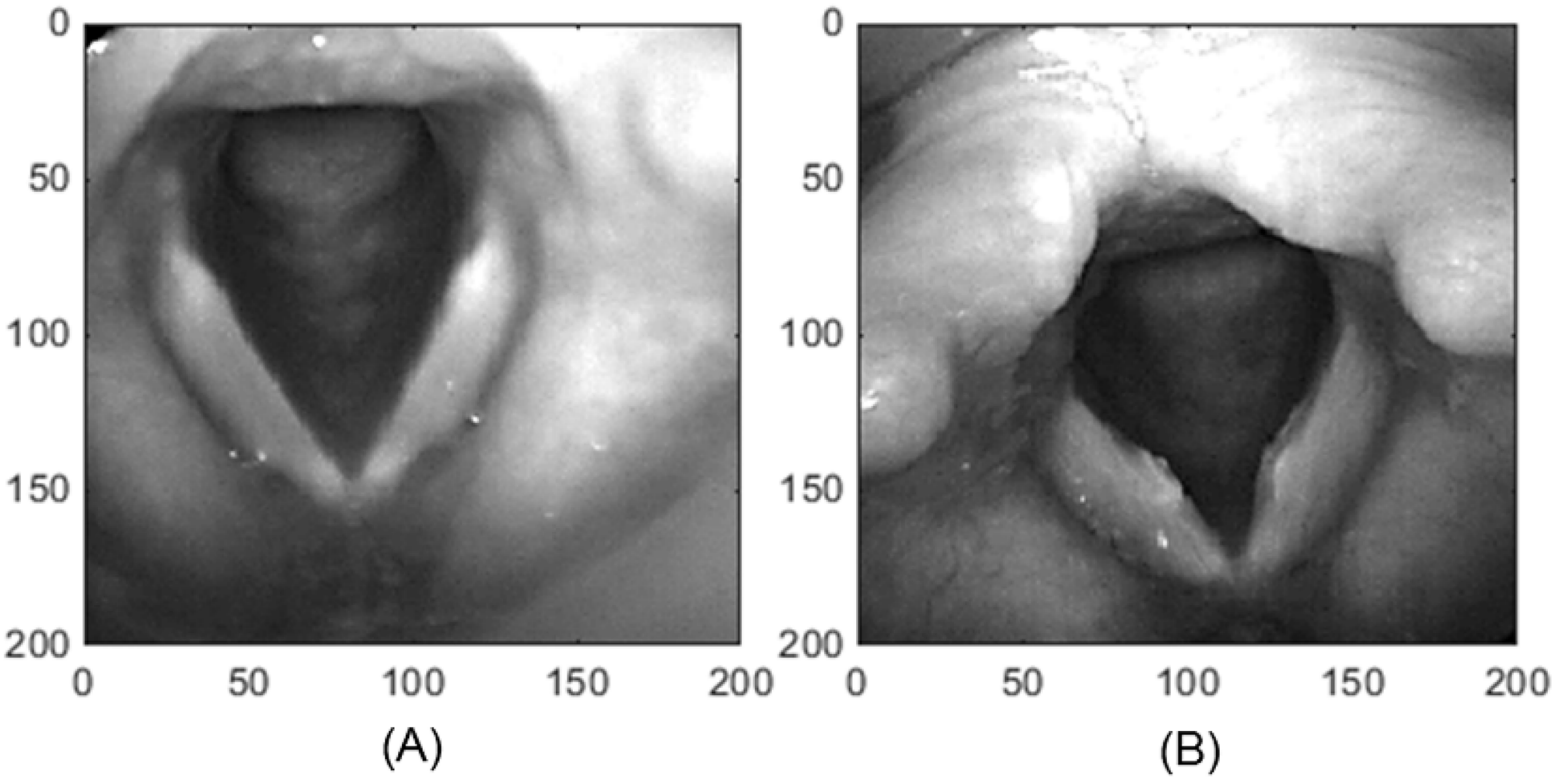
Vocal folds of a child without vocal fold nodules (A) and with bilateral vocal fold nodules (B). The x and y axis are in pixels.

Laryngeal imaging was captured using a grey scale high-speed camera (PENTAX digital Model 9710, Montvale, New Jersey) at 4000 frames per second with a spatial resolution of 512 x 256 pixels. A 70 degrees rigid endoscope was used to acquire the high-speed videos without the application of topical anesthetic to the oral mucosa. Participants were asked to sustain the vowel /i/ at conversational pitch and loudness levels for a maximum of 4.094 seconds. Simultaneous audio recording was captured at 50kHz. High-speed videoendoscopic images representing normal vocal fold and vocal fold nodules are displayed in Fig 1.

### Data Analysis

The high-speed videos were analyzed using a custom developed semi-automated image processing software called the Vocal Cord Analyzer (VCAbeta) [17]. A total of 30 consecutive cycles of steady-state phonation from each participant were selected for analysis. Left and right vocal fold displacements were extracted from the mid-membranous point of the vocal folds based on the medial glottal axis. The mid-membranous point of the vocal fold was selected for analysis since it corresponds to the region of maximum displacement along the length of the vocal fold [36]. The vocal fold displacements, however, can only be measured in pixels using the high-speed video system. The number of pixels displaced is dependent on the actual distance the vocal fold moves and the pixel density, which is dependent on the distance between the camera and the image plane. In order to limit the variability resulting from the unknown distances between the camera and the image plane on the computed features, robust metrics were created by normalizing the displacement by the glottal length. This results in units of glottal lengths for displacement and represents a geometric measure related to percentage of the vocal fold tissue displaced. Another potential source of variability in feature computation is the pitch of the voice. This is especially critical when comparing features across diverse populations [17]. Therefore in order to remove the influence of pitch on the computed features, the time scale for each glottal cycle was normalized by the glottal period. The normalization of the displacement waveforms used in this study permits quantification of relative displacements (in units of glottal length) and velocities (in units of glottal lengths per cycle). Features based on this normalized waveform cannot be used to infer actual velocities or displacements (without knowledge of actual glottal length); however, they do enable a quantitative assessment of the geometric properties of the vocal fold vibrations.

A total of 12 kinematic features were calculated to investigate the differences in vibratory motion between children with and without vocal fold nodules. Features based on displacement measures were computed for each vocal fold (left and right). The features reported in this study used the same approach as described in Patel et al (2015) [17]. This involved identifying 30 consecutive cycles of relatively stable pitch and volume. With the exception of the open quotient all features were computed for both left and right vocal folds from the mid membranous location of the vocal fold. The open quotient uses both right and left folds in its computation [37, 38]. Hence for the open quotient a single value was obtained for each cycle. For all the other features, the vocal fold with the greater value was used to represent a given cycle. The maximum value between the two vocal folds in each cycle was selected to capture the effect of higher impact stress, since higher impact stress is known to contribute to the development of vocal fold nodules. If the kinematic features were equal between the left and right folds, the average and the maximum values will be similar. Therefore, in cases where the folds are moving at different rates, the maximum value tracks the fold most likely to deliver the critical impact stress on closing, or experience the greater force during opening. After obtaining the values for each cycle, the mean was computed over all 30 cycles along with the cycle-to-cycle standard deviation. Means and standard deviations of the following kinematic features were computed: normalized peak displacement, normalized average opening velocity, normalized average closing velocity, normalized peak closing velocity, speed quotient, and open quotient.

**Fig 2:**
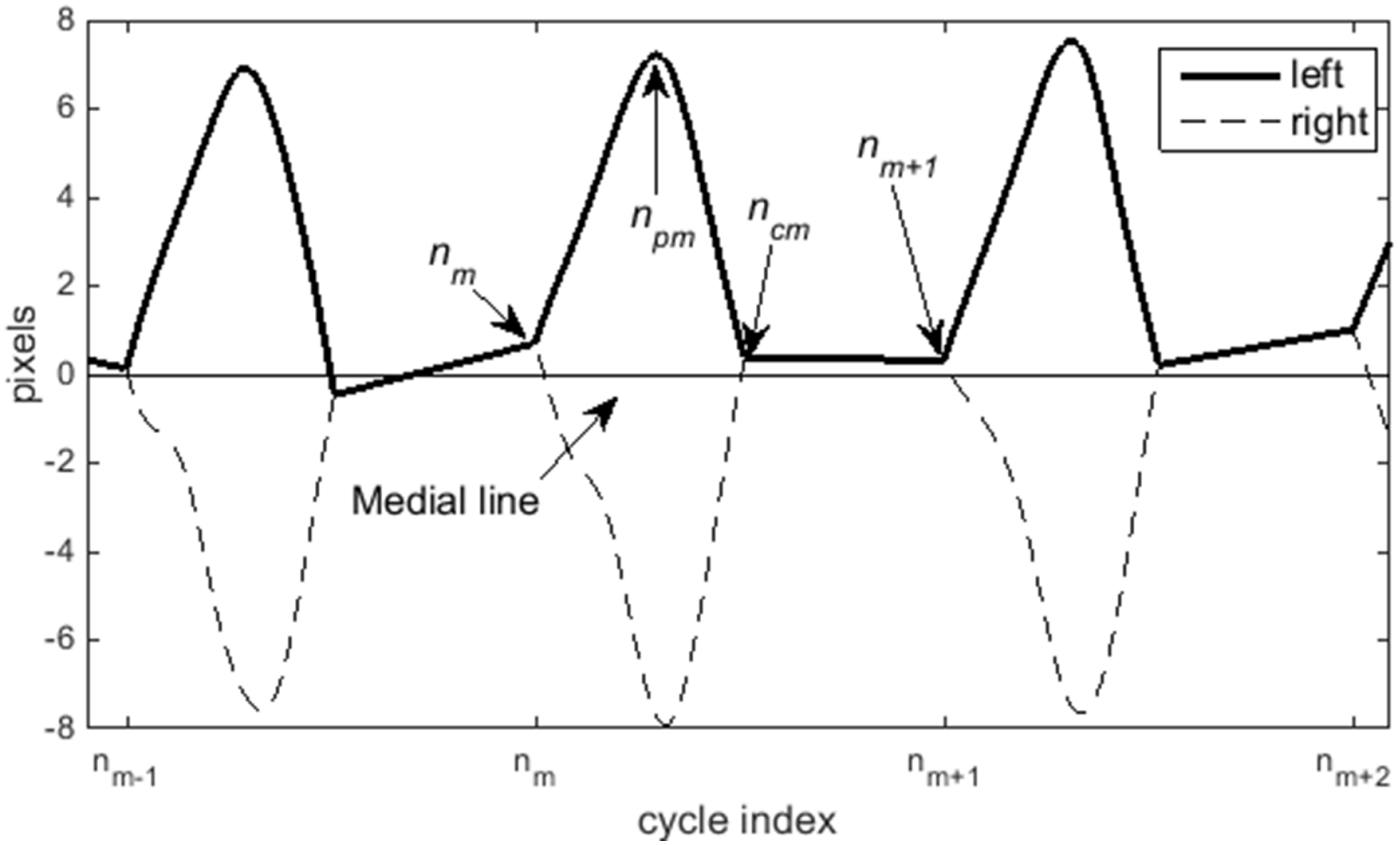
Illustration of critical points on displacement waveforms. Time-axis shows normalized glottal cycles and annotations show notation for denoted key events in the cycle, *n*_*m*_ = start opening phase, *n*_*pm*_ = peak displacement, *n*_*cm*_ = start of closed phase.

In order to explicitly describe these features let *L*[*n*] denote the discrete waveform representing the vocal fold edge displacement from the medial line in units of pixels. This displacement waveform was created through denoising to reduce errors from the quantization/pixilation effects and interpolation/extrapolation to improve locating the critical time instances, such as peak displacement and vocal fold opening/closing instances [17, 38]. The denoising and interpolation/extrapolation step is critical for obtaining stable estimates of features that rely on closing, peak, and opening events, such as the speed quotient, since a frame rate of 4000 Hz may only result in 5 to 6 samples per open phase, resulting in significant rounding error relative to the cycle length. The details for creating *L*[*n*] are presented in Patel et al (2015) [17]. An example of the left and right displacement waveform is shown in Fig 2 where the indices of critical samples are labeled on the plot, and the x-axis denotes the sequence of consecutive pitch-normalized cycles.

The **normalized peak displacement** [17] in terms of glottal length in each cycle is given by:

$$d_{p}[m]=\frac{\text{max}\left\{L[n]\right\}\;}{G_{L}}\text{ for }n_{m}\leq n<n_{m+1}$$ (1)

where *n*_*m*_ is the sample denoting the point before the folds separate to begin the opening phase for the *m*^th^ cycle (Fig 2), and *G*_*L*_ is the glottal length in pixels. The normalized peak displacement represents maximum lateral extension of the vocal fold. This normalization results in units of glottal lengths and relates to the percentage of the vocal fold tissue displaced in each cycle. This feature of normalized peak displacement has the advantage of being calculated from any video image (does not require special calibration to obtain actual distance) and allows for a consistent comparison between images and subjects.

The **normalized average opening velocity** [17] represents the relative velocity of the vocal fold during the opening phase and is given by:

$${\bar{V}}_{o}[m]=\frac{\left(L[n_{pm}]-L[n_{m}]\right)}{G_{L}}\left(\frac{n_{m+1}-n_{m}}{n_{pm}-n_{m}}\right)$$ (2)

where *n*_*pm*_ is the sample corresponding to the peak displacement in the *m*^*th*^ cycle (Fig 2). The numerator of the index fraction is the period the *m*^*th*^ cycle in samples and is the normalizing factor to remove the effect of pitch on the kinematic feature. Analogously, **the normalized average closing velocity** [17] is the relative velocity of the vocal fold during the closing phase of the glottal cycle and is given by:

$${\bar{V}}_{c}[m]=\frac{\left(L[n_{cm}]-L[n_{pm}]\right)}{G_{L}}\left(\frac{n_{m+1}-n_{m}}{n_{cm}-n_{pm}}\right)$$ (3)

where *n*_*cm*_ is the sample corresponding to the first point after closing in the *m*^*th*^ cycle.

The **peak closing velocity** [17] occurs when the fold is displaced by its greatest distance over a one sample interval during the closing phase. This is given by:

$$V_{pc}[m]=\frac{\text{max}\left\{L(n+1)-L(n)\right\}}{G_{L}}\;\left(n_{m+1}-n_{m}\right)\text{ for }n_{pm}\leq n<n_{cm}$$ (4)

The **speed quotient** represents the time required for the opening phase of the glottal cycle divided by the duration of the closing phase of the glottal cycle and is given by [17, 39]:

$$S_{}[m]=\frac{n_{pm}-n_{m}}{n_{cm}-n_{pm}}$$ (5)

The **open quotient** [39] is defined as the duration of the open phase (opening plus closing phase) of the glottal cycle divided by the total duration of one glottal cycle and is denoted as:

$$O_{}[m]=\frac{n_{cm}-n_{m}}{n_{m-1}-n_{m}}$$ (6)

The cycle features for the open and speed quotient are strictly based on the timing of the opening and closing events and do not include vocal fold displacement information (as features in Eqs 1–4 convey). These features relate to the relative time spent in each phase of the glottal cycle.

### Statistical Analysis

The Shapiro-Wilk test was used to investigate the distribution of the 12 dependent variables, which are the means and standard deviations of the features described in Eqs (1) through (6). The Kruskal-Wallis-H test was used to compare children with and without vocal fold nodules for non-normal population distributions, while an analysis of variance (ANOVA) was used to do the same for the normally distributed dependent variables. Bonferroni correction was applied to the alpha level (0.05/12 = 0.0042). The *p* values of ≤ 0.0042 were therefore considered significant. Cohen’s effect size (*d*) was computed for parameters with non-normal distribution; whereas partial eta squared effect size (ES) was computed for parameters with normal distribution. For Cohen’s *d* the value of 0.5 is considered a large effect, 0.3 is a medium effect, and 0.1 is a small effect [40, 41]. All analyses were performed using SPSS Statistics software Version 23.0.

## Results

The results of the Shapiro-Wilk test, shown in Tables 1 and 2, indicate that the means and standard deviations of all features were not normally distributed, except for the mean of the open quotient.

**Table 1:** Shapiro-Wilk test of normality for the mean values of the kinematic features.

Dependent Variables	Shapiro-Wilk Statistic (s-w)	*df*	*p*
Normalized peak displacement	0.923	40	0.009
Normalized average opening velocity	0.921	40	0.008
Normalized average closing velocity	0.930	40	0.016
Normalized peak closing velocity	0.935	40	0.024
Speed quotient	0.893	40	0.001
Open quotient	0.982	40	0.752

**Table 2:** Shapiro-Wilk test of normality for the standard deviation (*SD*) values of the kinematic features.

Dependent Variables	Shapiro-Wilk Statistic (s-w)	*df*	*p*
Normalized peak displacement_*SD*	0.851	40	0.000
Normalized average opening velocity_*SD*	0.867	40	0.000
Normalized average closing velocity_*SD*	0.804	40	0.000
Normalized peak closing velocity_*SD*	0.873	40	0.000
Speed quotient_*SD*	0.909	40	0.003
Open quotient_*SD*	0.800	40	0.000

**Fig 3:**
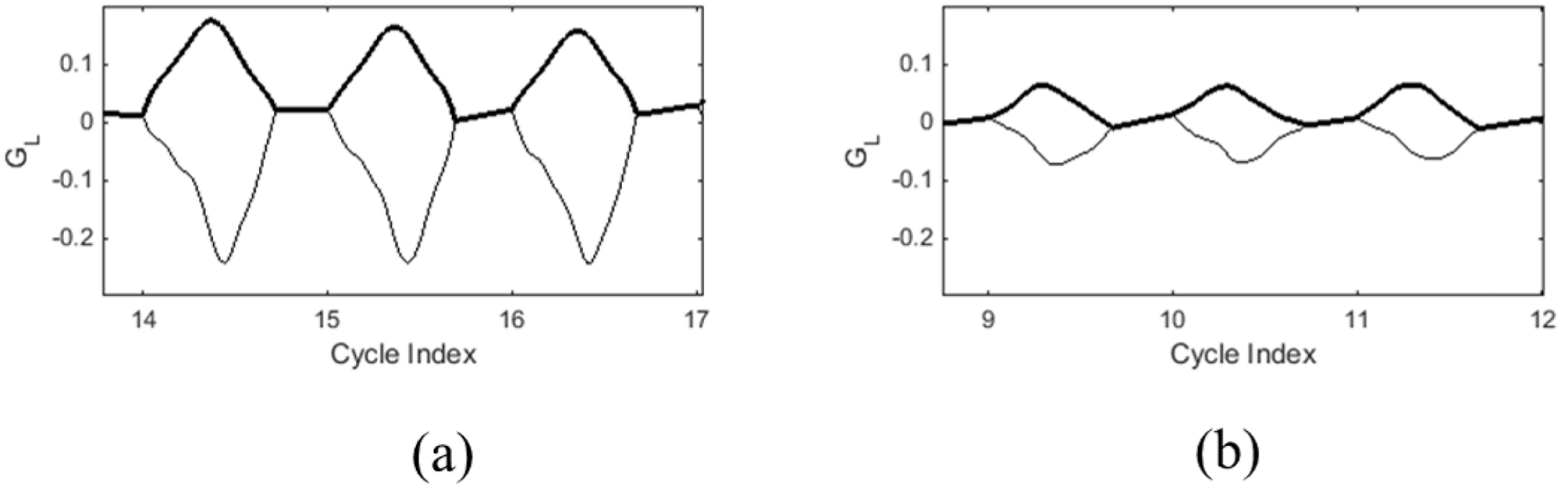
Right and left vocal fold displacement waveforms normalized by glottal length (*G*_*L*_) and cycle period for a (a) child with nodules and (b) without nodules. Darker line denotes the displacement of the left vocal fold. The thin line is the displacement of the right vocal fold.

Therefore, the Kruskal-Wallis-H test was applied to all features, except the open quotient, to test for significant differences between the two populations. Statistical significance was not obtained for the mean difference of all the features. For mean features, only the speed quotient showed a moderate effect size (Cohen’s *d*) of 0.358 (H(2) = 5.10, *p* = 0.02), with a mean rank of 16.32 for typically developing children and 24.68 for children with vocal fold nodules (Fig 3). A small effect size was observed for the normalized average closing velocity (H(2) = 2.86, *p* = 0.09, *d* = 0.267), normalized peak closing velocity (H(2) = 2.42, *p* = 0.12, *d* = 0.245), and normalized peak displacement (H(2) = 0.726, *p* = 0.39, *d* = 0.135) (Table 3).

**Table 3:** Comparison across mean values of the kinematic features between children with and without nodules.

Dependent Variables	Children without nodules		Children with nodules		*p* values	Effect size
	*Mean*	*SD*	*Mean*	*SD*		
Normalized peak displacement	0.105	0.045	0.120	0.056	0.39	0.135
Normalized average opening velocity	0.395	0.193	0.420	0.219	0.84	0.032
Normalized average closing velocity	0.349	0.167	0.461	0.208	0.09	0.267
Normalized peak closing velocity	0.656	0.319	0.808	0.333	0.12	0.245
Speed quotient	1.239	0.287	1.539	0.457	0.02	0.358
Open quotient	0.435	0.099	0.447	0.107	0.71	0.004

**Fig 4:**
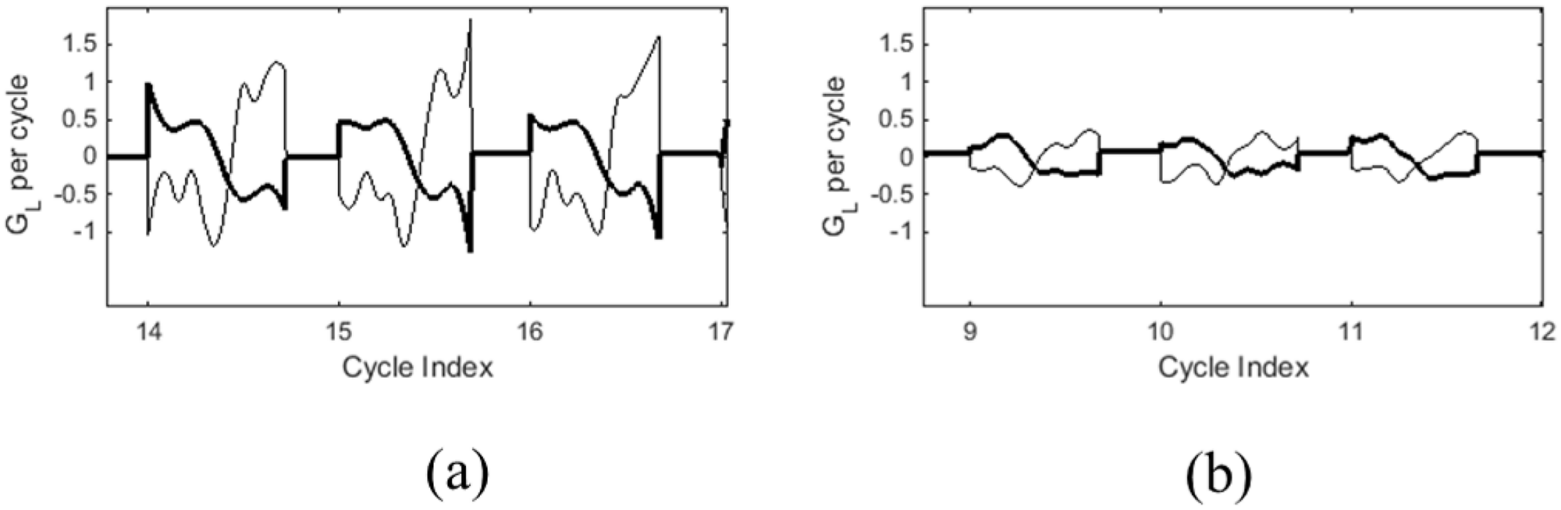
Normalized instantaneous velocities derived from the displacement waveforms in Fig 3 for (a) child with nodules and (b) without nodules. The darker line represents the normalized instantaneous velocity derived from the left vocal fold displacement. The thin line represents the normalized instantaneous velocity derived from the right vocal fold displacement. The y-axis is denoted by glottal length (*G*_*L*_) per cycle.

The Fig 3 compares sample displacement waveforms for a child with and without nodules illustrating the trends observed in Table 3. The Fig 3 reveals that the peak displacement is larger in the child with nodules (Fig 3a) compared to the typical child (Fig 3b). Though not as dramatic, it also shows a higher speed quotient for the child with vocal fold nodules. The mid-membranous vocal fold displacement in a child without nodules (Fig 3b) demonstrates a more even distribution between the opening and closing phase durations. This is consistent with the difference in the speed quotient feature where the children with nodules had a higher speed quotient. The Fig 4 shows the instantaneous velocities corresponding to the trajectories in Fig 3. These plots show more dramatically the relatively strong increases in rate during the closing phase of the glottal cycle for the child with nodules, whereas the normal child depicts smaller values of the normalized closing phase velocity. This is consistent with the trends of a higher normalized average closing velocity and a peak closing velocity for children with nodules.

Variability in the vocal fold vibrations were examined with the cycle-to-cycle standard deviations for each dependent variable across the two participant groups. There was no statistically significant difference for any of the cycle-to-cycle standard deviation variables. A moderate effect size was observed for the standard deviation of normalized average closing velocity (H(2) = 4.98, *p* = 0.03, *d* = 0.353), with a mean rank of 16.38 for typically developing children and 24.62 for children with vocal fold nodules. A small effect size was observed for the speed quotient_*SD* (H(2) = 1.76, *p* = 0.19, *d* = 0.209) (Table 4).

**Table 4:** Comparison across standard deviation of the kinematic features between children with and without nodules.

Dependent Variables	Children without nodules		Children with nodules		*p* values	Effect size
	*Mean*	*SD*	*Mean*	*SD*		
Normalized peak displacement_*SD*	0.006	0.003	0.007	0.006	0.90	0.019
Normalized average opening velocity_*SD*	0.034	0.023	0.038	0.025	0.71	0.060
Normalized average closing velocity_*SD*	0.035	0.018	0.050	0.030	0.03	0.353
Normalized peak closing velocity_*SD*	0.118	0.068	0.118	0.065	0.79	0.043
Speed quotient_*SD*	0.146	0.080	0.179	0.080	0.19	0.209
Open quotient_*SD*	0.025	0.011	0.025	0.017	0.54	0.096

## Discussion

Vocal fold nodules vary in size and result in mild to severe disturbances in the overall voice quality, which leads to a variety in glottal cycle displacement patterns that can be difficult to characterize visually over a population. Previous studies differentiating vocal function in children with and without nodules have used acoustic [15, 16] and aerodynamic [16] analysis. In terms of laryngeal imaging, attempts were made to develop a valid 4-point rating scale of nodule size and contour by presenting a series of 8 still videostroboscopic images [42]. Though useful, it does not show the impact of these nodules on vocal fold motion or potential kinematic patterns that can give rise and/or perpetuate the presence of vocal fold nodules. Empirical investigations of the impact of vocal fold nodules on vibratory motion are critical as clinical assessment of the nature of the lesions is based on visualization of structure and how it affects the vibratory motion. In addition, there is a paucity of literature regarding objective measurements of impaired vocal fold vibratory function from techniques of laryngeal imaging in the pediatric population. This study addresses this gap by quantitatively characterizing the kinematics of the fold motions between the pediatric population with and without nodules.

The goal of this initial study was to investigate features that quantify the effects of vocal fold nodules on vibratory function in children using high-speed videoendoscopy to determine population difference. The kinematic features used in this study were also used for comparing children and adults with normal voice [17]. These normalized kinematic features scale out differences due to variability from the uncalibrated pixel measurements as well as pitch differences, which can vary widely over the pediatric population. Popular features, such as open and speed quotient, work on a similar principle of scaling out what cannot be objectively measured (such as real distance), and also remove effects of pitch differences by taking ratios of glottal cycle sections. The motivation behind the additional features used here is to isolate critical phases of the vocal fold motion over the entire glottal cycle to understand more specifically, where differences between populations exist in the vocal fold kinematics. The interpretation of these features is primarily geometric; these features cannot be equated with absolute distance and velocity. The kinematic features essentially capture quantitatively what is observed qualitatively from the video. For example, absolute peak displacement is a distance measure that cannot be observed/measured in the actual video; however, by dividing the pixel distance by the glottal length, the normalized peak displacement can be interpreted as the portion of tissue displaced. Similarly, the normalized velocity based parameters associated with the opening and closing phases do not reflect absolute velocity but indicate relative velocities and how energy is distributed over the glottal cycle. These measures can be consistently computed across high-speed videos of the same patient at different times as well as over different populations of interest, and has the potential to serve as outcome measures before and after appropriate intervention.

We hypothesized that children with vocal fold nodules will have greater mean values of normalized peak displacement, normalized average opening velocity, normalized average closing velocity, normalized peak closing velocity, speed quotient, and open quotient compared to children without vocal fold nodules. The values for all of the above features were higher in children with vocal fold nodules, however, none of the comparisons achieved statistical significance. Due to the relatively small sample size, effect sizes were calculated to aid in revealing potentially differentiating measures that can guide future studies with larger samples. Compared to all the features, the speed quotient showed a moderate effect size between the two groups indicating speed quotient as a potentially salient feature compared to the other features.

The finding of increased speed quotient in children with vocal fold nodules is consistent with findings from high-speed films on one adult female with vocal fold nodules, where the speed quotient was 1.0 at the center of the nodules [37]. Changes in speed quotient are biomechanically attributed to changes in tension and air pressure [39]. The results here suggest that the presence of vocal hyperfunction results in increased speed quotient in children with vocal nodules and that this is primarily driven by greater forces in the closing phase. The later conjecture is inferred from the increased normalized closing velocities (small effect size). The study finding of increased speed quotient in children with vocal fold nodules appear consistent with current theories of pathogenesis of vocal fold nodules. Investigations from biomechanical studies have shown that for high local impact to occur, the vibratory amplitude of the ventral surface is typically large with incomplete adduction of the dorsal portion of the vocal folds [12]. Large speed quotient in children suggests a greater phase difference between the ventral and dorsal margins of the vocal folds, with either increase in the vibratory amplitude of the ventral margin or decrease in the amplitude of the dorsal margin [43]. Children with vocal fold nodules in this study demonstrated an increase trend in the normalized peak displacement, suggesting that the vibratory amplitude of the ventral margin was increased (if glottal lengths were similar); however this finding did not achieve statistical significance and had a small effect size. Future studies could investigate the relationship between the upper and lower margins of the vocal fold through techniques of finite element modeling or with the use of laser endoscopy coupled with high-speed videoendoscopy [33].

Spatial features of normalized average closing velocity, normalized peak closing velocity, and normalized peak displacement demonstrated a mild effect size for increase in children with nodules compared to typically developing children. The increase in these values is consistent with existing theories of vocal hyperfunction and impact stress leading to the development of vocal fold nodules [1, 2, 14, 44]. Individuals with vocal nodules often have greater vocal intensities, thereby requiring greater subglottal pressure to vibrate the vocal folds due to increased glottal resistance [45]. A greater subglottal pressure generally results in a sharper closing phase of the glottal cycle; a finding which was observed in the study but was not statistically significant. The lack of statistical significance of these features in the present study may be due to a number of reasons including reduced sample size, high-inter subject variability, and lack of sensitivity of the parameters to capture the differences between the two groups. Further, the lack of statistical significance in the spatial features could also be due to the variability in the size of the nodules in the experimental group creating greater variabilities in the opening and closing phase trajectories as indicated by the higher standard deviations for each feature except the normalized peak closing velocity.

The speed quotient appears to have the best effect size for separating the populations, followed by the normalized average and peak closing velocities. The speed quotient is not directly impacted by variabilities in the open quotient or peak displacements. On the other hand, normalized closing velocities are impacted by the open quotient and the peak displacements. These additional variability resulting from these dependencies coupled with their weaker differences between the populations, explains why the normalized closing values had smaller effect sizes relative to the speed quotient. However, these trends show that the difference in the speed quotient between the two groups was likely driven by the faster normalized closing velocities. In comparing the average normalized opening and closing velocities, there was a small difference in the normalized average opening velocity and a larger difference in the average closing velocity. Table 3 indicates a similar normalized opening velocity between both groups with a population mean difference of 0.03 and a combined population standard deviation of 0.29 (sum of variances and square root), while the normalized average closing velocity shows a greater difference of 0.11 and a combined standard deviation of 0.27, which suggests that the faster normalized closing velocity is driving the significant difference observed in the speed quotient.

We hypothesized that children with vocal fold nodules will have greater temporal variability compared to children without vocal fold nodules. Higher temporal variability was expected to result in large mean values of normalized peak displacement_SD, normalized average opening velocity_SD, normalized average closing velocity_SD, normalized peak closing velocity_SD, speed quotient_SD, and open quotient_SD compared to children without vocal fold nodules. A moderate effect size was observed for only the temporal feature of average closing velocity indicating that the variability in the average closing velocity was a salient feature in differentiating children with and without nodules compared to the other temporal features. High temporal variability in the normalized average closing velocity suggests reduced stability of the closing phase compared to the opening phase. This high variability also suggests that intermittent cycles have very high normalized closing velocities with relatively high impact stress. The increased cycle-to-cycle variability in normalized average closing velocity is in contrast to the normal population, which shows a stronger clustering about the mean. The actual source of the variability could be driven by the presence of the nodule resulting in a greater non-uniformity of the distribution of tissue mass and airflow during the phonation process, or it could be a pattern of phonation that was present before the nodules were formed. The findings from Patel et al (2015) [17] showed that vocally normal children had a greater standard deviation in average closing velocity relative to the adult population with stiffer vocal folds. The normal children in this study match the normal pediatric population in Patel et al (2015) [17] for the standard deviation of the normalized average closing velocity (both at .035), demonstrating the likelihood that this feature is more repeatable and useful as a distinguishing feature for both normal children and adults, as well as between vocally normal children and children with vocal nodules.

The temporal features of normalized peak displacement, normalized average opening velocity, normalized peak closing velocity, and open quotient demonstrated a non-significant increase in children with nodules compared to typically developing children, however did not have a moderate/small effect size. The lack of statistical significance and moderate effect size may be due to a number of reasons including reduced sample size, high-inter subject variability, and lack of sensitivity of the parameters to capture the differences between the two groups.

The present study is limited in that only one type of lesion was investigated. Hence, it must be emphasized that the findings cannot be generalized to other mass lesions encountered in children. However, the differences in the speed quotient and the variability in the closing phase feature in the normal and the disordered group is encouraging as it could serve as the basis for future investigations in this area where other disorders could be differentiated.

### Methodological Considerations

The methods used in this work were especially developed to characterize vibratory motion in pediatric populations, which are characterized by shorter glottal cycles and greater variability in pitch and distance between the camera and image plane (due to developmental stages). While scaling by glottal length and pitch addressed the later issue, the shorter glottal cycles require methods to mitigate the potential instabilities and higher error rates due to the limited number of samples per-cycle. The features in this paper were computed from a 4000 fps video, which for the short vibratory periods in children, could potentially result in high errors for each feature estimate, especially those that use time interval estimate in the denominators of the computations (e.g. speed quotient). A direct approach of increasing the sampling rate can address this error but at the cost of increased lighting and hardware requirements. The sensors in the camera integrate the light falling on them for a capture time [46]. This integration over time helps in capturing more photons per frame and reduces the sensor noise, resulting in more reliable edge detection performance. If the sampling frequency is increased, less integration takes place and more sensor noise is present, resulting in greater noise in the image. Hence, an increase in frame rate alone does not guarantee reduced errors in feature estimates.

Given the quasi-periodic motion of the vibrating folds, denosing (smoothing), interpolation, and extrapolation can be used to obtain feature estimates with lower error, relative to simply using values from the points on the sampling grid and pixel positions [17, 38]. For the subjects used in this study, the children without nodules had an average pitch of 287 Hz (SD = 40) and those with nodules had an average pitch of 268 Hz (SD = 30). For a 287 Hz pitch, the error from rounding time points of key events in the vibrating folds, such as opening, maximum displacement, and closing can be as high as 7.2%. This error can translate in to large feature error estimates when it affects both the numerator and denominator of the computation. For example, a subject with a pitch of 287 Hz and symmetric open and speed quotients, the error from rounding the opening and closing event times to near the nearest sample grid point can result in a maximum error of 0.25 ms. If the maximum displacement time is rounded to the nearest sample grid point (i.e. pixel with maximum displacement), a maximum error of 0.125ms can occur. For speed quotient the closing phase time interval is in the denominator of the computation and opening phase in the numerator. In the worst case the opening phase is rounded up and the closing phase is rounded down for the maximum possible error, resulting in an 87% error in the estimate of the speed quotient. Assuming a uniform distribution on the rounding error (as typically done) the speed quotient estimate will have an average error of 35%. This error can be reduced by using interpolating/extrapolating to estimate the critical time points on a higher resolution grid.

For normalized features quantities, errors also occur from rounding locations to the nearest pixel locations. While the video image may have a high resolution in the total image plane, only the area covered by the vocal fold motion is critical. In most cases this ranges from 6 to 16 pixels. When the actual location is rounded to the pixel center, a quantization error occurs and results in a non-smooth trajectory for the fold. This will have an impact on any instantaneous measures relying on the derivative of the waveform and maximum displacement, and will certainly have a negative impact on the interpolation/extrapolation performed on the sampled points as demonstrated in Unnikrishnan et al (2012) [37].

Both the errors described above were addressed by the use of denoising, interpolation, and extrapolation schemes introduced in Unnikrishnan et al (2012) [38]. All features presented here show a cycle-to-cycle standard deviations at approximately an order of magnitude less than the mean values suggesting stability of the computations resulting from the smoothing and interpolation. Experiments are currently being developed and performed in our lab to more directly assess the limits of these techniques for estimating features from lower sampling rates of 4000fps compared to 5333fps, 8000fps, and 16,000fps.

### Conclusions

The kinematic feature analysis shows children with nodules demonstrate primary difference in the closing phase of the glottal cycle compared to the opening phase. Children with nodules demonstrate similar opening phase kinematics. Prominent differences are reflected in the speed quotient and variability in the normalized average closing velocity, which are both result from reduced durations of the closing phase. The moderate effect sizes for these features suggests the need for experiments with large number of subjects, focusing on features related to the closing phase for characterizing the presence and severity of vocal fold nodules.

## Supporting Information

S1 File Individual subject data. (XLSX)
